# Revising Career Goals on LinkedIn: The Role of Career-Related Social Comparison and Goal Discrepancy

**DOI:** 10.3390/bs16081271

**Published:** 2026-07-24

**Authors:** Maria Vogt, Elke Rohmann, Hans-Werner Bierhoff, Phillip Ozimek

**Affiliations:** 1 Department of Psychology, Ruhr University Bochum, Universitaetsstrasse 150, 44801 Bochum, Germany; maria.vogt@ruhr-uni-bochum.de (M.V.); elke.rohmann@rub.de (E.R.); hans.bierhoff@rub.de (H.-W.B.); 2 Department of Humanities, Vinzenz Pallotti University, Pallottistrasse 3, 56179 Vallendar, Germany

**Keywords:** LinkedIn, career-related social comparison, career goal discrepancy, career goals, upward goal revision, downward goal revision, career centrality

## Abstract

Professional networking platforms provide opportunities for career development but may also encourage career-related social comparison. This study examined whether the relationship between LinkedIn use and career goal revision is explained by career-related social comparison and perceived career goal discrepancy. Data from 188 LinkedIn users were analyzed using correlation and serial mediation analysis. More intensive LinkedIn use was associated with more frequent career-related social comparison, which was related to greater perceived career goal discrepancy and, in turn, stronger career goal revision. The proposed serial mediation model was supported. Post hoc analyses revealed differences between upward and downward career goal revision. Whereas the serial mediation pathway explained downward goal revision, upward goal revision was directly associated with LinkedIn use and was not explained by the proposed mediators. Career centrality was positively associated with career-related social comparison but did not influence the proposed relationships. The findings highlight LinkedIn as a context for career-related self-evaluation and goal regulation.

## 1. Introduction

Since its founding in 2003, LinkedIn has provided a digital space where individuals can build professional relationships, learn about career opportunities, and strategically drive their professional development (Paliszkiewicz & Madra-Sawicka, 2016; Zide et al., 2014). The platform has established itself as an effective tool in everyday professional life (McCabe, 2017). With over 1.9 million feed posts being viewed per minute, it becomes clear just how intensively LinkedIn is used (LinkedIn, n.d.). At the same time, the constant visibility of professional achievements creates an environment that invites users to compare careers.

Comparisons between one’s own realistic offline self and the idealized online selves of others often turn out negatively (Vogel et al., 2014) and can foster dissatisfaction with one’s own career situation (Fukubayashi & Fuji, 2021). Some people set even more ambitious goals and increase their efforts (Creed & Hood, 2015) to keep pace with seemingly more successful individuals. Others, however, perceive their previous goals as difficult to achieve or unrealistic and subsequently adjust them downward (Theobald et al., 2025).

When there is a perceived discrepancy between one’s career goals and actual personal progress toward achieving them, goals that were previously viewed as motivating trigger disappointment and stress (Creed & Hood, 2015; Praskova & McPeake, 2022). It has been repeatedly demonstrated that this perceived career goal discrepancy influences the revision of goals (Donovan & Williams, 2003; Theobald et al., 2025; Tolli & Schmidt, 2008). The role of career-related social comparison has so far been examined only marginally, yet it may be precisely this factor that raises awareness of such discrepancies.

While social comparison processes on social networks such as Facebook or Instagram have already been studied extensively (Verduyn et al., 2020; Vogel et al., 2014), the specific context of career-oriented networks has remained largely unexplored, even though the use of career-oriented platforms has significantly increased in the past few years (Sonnenberg, 2026). Although Brandenberg et al. (2019) provided initial evidence of a relationship between the use of professional networks and social comparison in their study on XING, an explicit career-related focus has been lacking to date. Career-related social comparison has been addressed only once so far as an independent construct in the literature (Fukubayashi & Fuji, 2021). This underscores the gaps in the current state of research. The same applies to research on goal revision. Established goal theories, such as the one by Locke and Latham (2002), provide fundamental insights into how goals are adjusted; however, they are primarily applied in the context of education and sports (Theobald et al., 2025). Career goals, in contrast, are significantly more complex and long-term in nature (Hu et al., 2017), which is why the transferability of these earlier findings to the career context is limited. Unlike educational or athletic goals, career goals typically develop over extended periods, involve greater uncertainty, and are influenced by external labor market conditions and social evaluations. Moreover, LinkedIn continuously exposes users to curated information about other people’s professional achievements, creating a unique context in which career goals may be repeatedly evaluated and adjusted in response to career-related social comparison.

Although previous studies have linked professional networking platforms to social comparison (e.g., Brandenberg et al., 2019), career-related social comparison to career dissatisfaction (Fukubayashi & Fuji, 2021), and career goal discrepancy to goal revision (Praskova & McPeake, 2022), no study has integrated these mechanisms into a single process model explaining how LinkedIn use may ultimately relate to career goal revision. Therefore, this study addresses this research gap. Its aim is to expand the currently fragmented research on social comparisons on LinkedIn and to embed it within the career context. It examines the extent to which LinkedIn use is associated with the revision of individual career goals and whether this relationship specifically unfolds via career-related social comparison and the perceived career goal discrepancy as serial mediators. The overarching research question is: What significance does LinkedIn play as a context for social comparison in the evaluation and revision of individual career goals? Additionally, an exploratory research question examines the extent to which individual career centrality influences the relationship between LinkedIn use and career-related social comparison. Career goal revision may involve either lowering previously pursued goals (downward revision) or adopting more ambitious goals (upward revision). Although the present study initially examines goal revision as an overall construct, these two forms are explored separately in post hoc analyses.

## 2. Theoretical Background

### 2.1. LinkedIn as a Social Network

There is a wide variety of social networks that differ in terms of their functions and structure addressing different target groups (Aichner et al., 2021; Papacharissi, 2009; Röll, 2014). Therefore, the intended use varies depending on the platform, ranging from private communication to corporate communication and professional networking (Aichner et al., 2021). This also determines the perceived value of a platform by its users (Papacharissi, 2009).

LinkedIn is the world’s largest professional networking platform with more than 1.3 billion members. It is primarily used for networking, career development, and recruitment (Florenthal, 2015; LinkedIn, n.d.; Marshall, 2022; Zide et al., 2014). Members can search for jobs, while companies can post job openings and review potential candidates (Sievers et al., 2015). This allows companies to quickly access a large number of potential candidates and obtain comprehensive information about them through their public profiles (Zide et al., 2014).

LinkedIn profiles are highly standardized and focus primarily on education, work experience, and professional achievements (Sievers et al., 2015; Utz, 2016). Users typically present an idealized professional identity while avoiding the communication of failures and setbacks (Paliszkiewicz & Madra-Sawicka, 2016; Tran & Diep, 2025; Van Dijck, 2013; Weiss & Glück, 2024). Consequently, the platform provides a particularly salient context for career-related social comparison.

### 2.2. Social Comparison on Social Media

Social comparison refers to the tendency to evaluate one’s own abilities, opinions, and achievements by comparing them with those of others (Festinger, 1954). Comparisons may serve self-evaluation, self-enhancement, or self-improvement and can be directed upward or downward depending on the comparison target (Buunk & Gibbons, 2007; Wood, 1989). Frequent LinkedIn use has been associated with both career-related benefits and negative psychological outcomes (Bilderback, 2025; Davis et al., 2020; Wright et al., 2021).

Who compares themselves and how often can vary greatly. Research shows that the tendency toward social comparison is a disposition, with a higher degree of this disposition being associated with more frequent social comparisons and specific characteristics, such as a certain degree of self-doubt (Buunk & Gibbons, 2007; Gibbons & Buunk, 1999). Social comparisons have repeatedly been associated with lower well-being, self-esteem, and mental health, particularly when upward comparison processes dominate (Chen et al., 2023; Li, 2019; Wang et al., 2017).

In the context of social media, it is evident that as the intensity of use increases, so does the frequency of social comparisons (Chen et al., 2023). This can be attributed to two distinctive features of social networks. First, users can simultaneously compare themselves to a significantly larger number of people; second, they have access to more extensive information about the (potentially unknown) comparison targets (Vogel et al., 2015) than would be the case with acquaintances in their immediate social circle. In particular, the mere consumption of content, referred to as passive use, leads to upward comparisons and feelings of envy (Verduyn et al., 2020; Wang et al., 2017).

On social media, social comparisons span a wide range of topics, from physical appearance to popularity and social status to significant life events (Faelens et al., 2019; Verduyn et al., 2020; Vogel et al., 2014). In a professional context, however, social comparisons primarily relate to career success and professional position (Fukubayashi & Fuji, 2021; Heslin, 2003). This context-specific comparison, specifically called career-related social comparison, is the focus of this study. Heslin (2003) demonstrated that the evaluation of one’s own career success is based predominantly on the expectations and outcomes of other people. In total, 68% of respondents relied on comparison-based criteria when evaluating their success, such as income comparisons with their own age cohort. Furthermore, recent findings indicate that social comparisons are associated with professional goals. For instance, among young adults (18–35) frequent career-related social comparisons were linked to higher career ambitions (Sharma, 2025).

On LinkedIn, public success metrics such as likes and the highlighted profile content reinforce career-related comparison behavior (Bilderback, 2025) by facilitating the unconscious application of the availability heuristic, in which evaluations are based on the most readily available information (Chou & Edge, 2012). Since LinkedIn is primarily used to share positive professional developments and achievements (Oliver et al., 2024), it is primarily this positive content that is mentally present during comparisons (Chou & Edge, 2012). This fosters the perception that others are more successful and happier (Chou & Edge, 2012). Comparisons with profiles that appear more successful trigger the feeling among users that they are less perfect, competent, or successful than others (Chou & Edge, 2012; Ozimek & Bierhoff, 2016). Therefore, users critically question their own career resulting in professional frustration (Fukubayashi & Fuji, 2021).

Career-related social comparison differs from general social comparison because it focuses specifically on evaluating one’s professional achievements, career progress, competencies, and occupational status rather than broader domains such as appearance, lifestyle, or social relationships. Because career goals are inherently linked to evaluations of professional success, career-related social comparison is theoretically expected to be more directly related to career goal discrepancy and subsequent goal revision than general social comparison.

Taken together, LinkedIn provides a unique environment in which users are repeatedly exposed to selectively positive indicators of professional success. These career-related cues may encourage social comparison, which in turn can increase awareness of discrepancies between one’s current career situation and desired career goals. Such discrepancies may ultimately motivate individuals to revise their career goals. The following sections elaborate on these latter processes.

### 2.3. Career Goals

To assess their own careers, individuals typically rely on specific career goals, which they use to evaluate their professional progress. Career goals refer to a person’s long-term professional ambitions and aspirations (Ma et al., 2025; Sharma, 2025). They serve as central motivators for choosing a career path (Ma et al., 2025) and promote goal-oriented planning, which in turn facilitates the achievement of set goals (Greenhaus et al., 1995; Widyowati et al., 2024). Furthermore, career goals contribute to a concrete vision of the future (Greenhaus et al., 1995) fostering an optimistic career attitude (Widyowati et al., 2024). Given the variety of career options (Creed & Hood, 2014), choosing a career goal represents one of the most significant challenges in life (Aydemir & Bayram, 2025). Note that career goals are underrepresented in recent research because only a few studies on this topic have been published in the past decade (Mulyana et al., 2025).

#### 2.3.1. Career Goal Discrepancy

The theoretical framework for analyzing goal-setting processes in a career context is provided by various goal-setting models including the goal-setting theory of Locke and Latham (2002), and Bandura’s (1991) social cognitive learning theory. Goal-setting theories assume that individuals evaluate their progress by comparing their current state with a desired goal state (Locke & Latham, 2002). When discrepancies emerge between goals and perceived progress, individuals may adapt their behavior or revise their goals accordingly (Theobald et al., 2025; van Lent, 2019). According to goal-setting models, this process is structured as follows:

- First, a goal is set or given, either consciously or unconsciously.
- This is followed by the identification and evaluation of the deviation between the goal and the current performance, whereby this discrepancy is positive, negative, or nonexistent (Creed et al., 2015; Theobald et al., 2025). The potential career goal discrepancy refers to the perceived deviation between a desired career goal and the progress made toward that goal (Creed & Hood, 2015). Thus, the desired future situation is compared with the current one. Perceived discrepancies in the career context can arise when individuals assess their current level of development in key areas such as performance, skills, or effort in relation to their career goals (Widyowati et al., 2024). What a perceived discrepancy causes on an emotional level depends on its direction. A negative discrepancy occurs when the performance is lower than the goal; a positive discrepancy arises when the performance exceeds the goal (Theobald et al., 2025). The two directions are in contrast. While positive discrepancies primarily result in satisfaction, negative discrepancies lead to increased frustration, disappointment, a loss of perceived meaningfulness and controllability of the situation, as well as feelings of failure when there is a lack of confidence in achieving the goal (Creed et al., 2015, 2021; Praskova & McPeake, 2022). However, according to social cognitive learning theory (Bandura, 1991), higher goals can also motivate to peak performance, provided they are perceived as achievable (Tolli & Schmidt, 2008). Accordingly, some people consciously set goals that exceed their current level of performance and thereby create a negative discrepancy to strive for continuous self-improvement (Theobald et al., 2025).

As individuals pursue their career goals, they continually assess whether the goal is appropriate and how successful they are at making progress (Creed et al., 2015; Widyowati et al., 2024). In this context, feedback is obtained from two different sources: internal self-referential criteria and external sources, such as social comparisons (Latzke & Schiffinger, 2012; Widyowati et al., 2024). The comparison of one’s own progress with the perceived standards reveals discrepancies (Creed & Hood, 2015), which in turn serve as feedback, as the extent of the deviation provides an estimate of the progress that is still needed (Widyowati et al., 2024). The resulting cognitive dissonance encourages the adoption of strategies to reduce this discrepancy, such as changing goal-directed behavior or revising goals.

In the present model, career-related social comparison is conceptualized as an external source of evaluative information. Comparing one’s own career with the perceived achievements of others provides a reference standard against which current career progress can be evaluated. Consequently, social comparison is assumed to precede the perception of career goal discrepancy, although alternative temporal sequences remain possible and should be examined in longitudinal research.

#### 2.3.2. Revision of Career Goals

Unfortunately, not much is known about how people actually revise their goals. In their meta-analysis, Theobald et al. (2025) identified merely 28 articles comprising 37 studies published between 1940 and 2022 that explicitly measured goal revision, with the majority stemming from educational psychology (64%) primarily examining university students (92%). This analysis revealed as expected that people raise their goals after success and lower them after failure. This process of upward and downward goal revision constitutes a dynamic process in which self-set goals are evaluated and adjusted in response to goal attainment or goal failure. Note that downward revision refers to striving for a less demanding, less prestigious, and more realistic goal, while upward revision involves setting a more ambitious, potentially more prestigious goal (Hu et al., 2017, 2019).

Studies on upward and downward goal revision have so far primarily focused on students and athletic performance (van Lent, 2019). A study by Donovan and Hafsteinsson (2006) examined the goal revision behavior of athletes. The findings showed that individuals whose performance fell short of their goals adjusted their goals downward, with the extent of the adjustment being proportional to the magnitude of the discrepancy. Conversely, individuals who exceeded their goals raised them and set more ambitious goals for the future, although this occurred less frequently (Donovan & Hafsteinsson, 2006). Further studies confirm this pattern (Theobald et al., 2025; Widyowati et al., 2024). The magnitude of the perceived discrepancy is the primary predictor for estimating the extent of goal revision (Donovan & Hafsteinsson, 2006); empirically, a strong correlation is observed (r = 0.51; Theobald et al., 2025). In the case of success, the discrepancy explains nearly 50% of the variance in upward goal revision, with a significant overachievement leading to a greater increase in subsequent goals (Donovan & Hafsteinsson, 2006). In the case of failure, it was found that greater career goal discrepancies were associated with a stronger intention towards downward goal revision (Widyowati et al., 2024).

In a career context, a significant causal relationship between career-related stress and downward goal revision was demonstrated (Hu et al., 2017). The longitudinal study of the student sample showed that individuals with higher career-related stress adjusted their goals downward over time but did not abandon them (Hu et al., 2017). Consistent with this, Praskova and McPeake (2022) demonstrated that young adults who experience increased career-related stress due to a career goal discrepancy respond by lowering their goals and reducing the effort they invest in their current career goal.

Alternative approaches distinguish between goal disengagement and reengagement when goals become unattainable (Wrosch et al., 2003). However, because career development typically involves adjusting rather than abandoning goals, the present study focuses on upward and downward goal revision (Creed & Hood, 2014; Hu et al., 2017).

### 2.4. Career Centrality

People who place greater importance on their career goals show a stronger desire to achieve them (Hu et al., 2018). This perceived importance of career goals varies among individuals (Ji et al., 2025). While for some people professional advancement forms a central aim in life, for others, career holds a lower priority. This individual difference is captured by the construct of career centrality which refers to an individual’s perception of the importance of their career in their own life (Paullay et al., 1994).

A conceptually related construct is work centrality. The key difference between the two concepts lies in the fact that career centrality focuses on career and thus professional advancement, whereas work centrality captures the general relevance of work in a person’s life. Due to this conceptual overlap and the very limited empirical research on career centrality to date, the more extensive research on work centrality is drawn upon for theoretical classification (Erdogan et al., 2018; Ji et al., 2025). This distinction is particularly relevant in the context of LinkedIn. Whereas work centrality reflects the general importance of work in an individual’s life, career centrality specifically captures the importance attached to long-term career development, professional advancement, and career success. Because LinkedIn is primarily designed to support networking, career progression, and professional identity construction rather than work itself, career centrality represents the more appropriate construct for the present study.

## 3. Research Questions and Hypotheses

As the leading professional network, LinkedIn is geared toward networking and career development (Florenthal, 2015; Zide et al., 2014) and motivates users to actively shape their careers (Gerard, 2012). The platform is thus used within a career-related context that can influence expectations regarding one’s own professional trajectory. This suggests that LinkedIn use not only triggers cognitive processes at the career level but may also prompt intentions to adjust behavior. This leads to the following hypothesis:

**H1.**  *There is a positive association between the intensity of LinkedIn use and the revision of career goals.*

It has been empirically demonstrated that social networks elicit social comparison (Verduyn et al., 2020; Vogel et al., 2015). For example, research on the platform Instagram has shown that more frequent use is associated with a higher degree of social comparison (Fagundes et al., 2020). Due to its structural focus on professional self-presentation and the continuous exposure to career-relevant information, LinkedIn offers a highly suitable context for career-related comparisons. This is supported by findings regarding the similar professional network XING, which show that increased usage especially results in skill comparisons (Brandenberg et al., 2019). Furthermore, it has been demonstrated that viewing career-related posts on social networks is associated with career-related social comparisons (Fukubayashi & Fuji, 2021). Therefore, the following hypothesis is derived:

**H2.**  *There is a positive association between the intensity of LinkedIn use and career-related social comparison.*

Social comparisons enable individuals to perceive discrepancies (Latzke & Schiffinger, 2012) by using others as a benchmark for evaluating their own performance (Festinger, 1954). An experimental study by Haferkamp and Krämer (2011) demonstrated that this holds true in the context of social networks. For men, comparing themselves to more professionally successful profiles led to an increased career goal discrepancy, whereas this was not the case with less successful profiles. The information gained through social comparisons can thus heighten awareness of discrepancies between one’s current career status and one’s desired goals. Studies on the impact of feedback on career goal discrepancy fit into this context. Social comparison in the professional context can be understood as a form of feedback that provides insights into current career progress (Hu et al., 2018). Studies show that a higher level of negative feedback is associated with a greater career goal discrepancy (Creed et al., 2015; Hu et al., 2018), with both constructs correlating positively (*r* = 0.55) (Praskova & McPeake, 2022).

Furthermore, it has been proven that career-related social comparisons on social media trigger professional frustration (Fukubayashi & Fuji, 2021) which manifests itself in the feeling of being stuck in one’s career or not making progress. The conclusion is that career-related social comparison as a source of feedback is related to career goal discrepancy. The corresponding hypothesis is:

**H3.**  *There is a positive association between career-related social comparison and the perceived career goal discrepancy.*

Multiple studies have examined the relationship between perceived goal discrepancy and goal revision processes (Donovan & Hafsteinsson, 2006; Donovan & Williams, 2003; Tolli & Schmidt, 2008). Discrepancies between the current state and the target state act as regulatory signals indicating a need for revision. In addition, in the career context, it has also been shown that perceived deviations from the desired career progress are associated with changes in career goal setting (Praskova & McPeake, 2022; Widyowati et al., 2024). Based on these findings, the following hypothesis is proposed:

**H4.**  *There is a positive association between perceived career goal discrepancy and career goal revision.*

In summary, more intensive use of LinkedIn is associated with career-related social comparison, which is linked to the perception of career goal discrepancies, and revision of career goals. Therefore, the following serial mediation path model is proposed:

**H5.**  *The positive association between the intensity of LinkedIn use and career goal revision is serially mediated by career-related social comparison and career goal discrepancy.*

**Figure 1:**
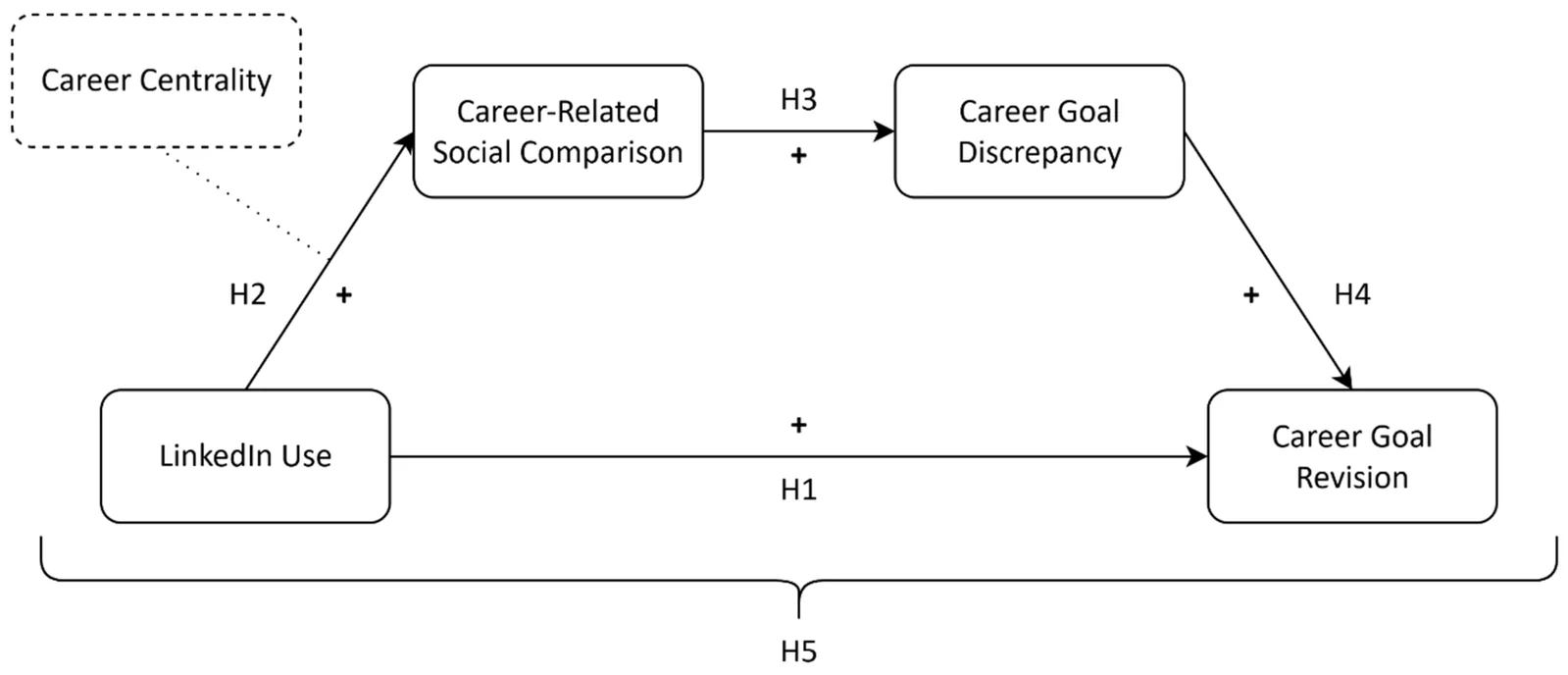
Overview of the hypotheses. Note. Assumed positive relationships are indicated by a plus sign. Hypothesis 5 assumes serial mediation and thus spans the entire model. The dashed line represents the exploratory analysis.

A visual overview of the hypotheses is presented in Figure 1.

The subjective meaning of career varies greatly from person to person. Festinger (1954) already postulated that the urge to compare oneself is particularly pronounced if a high subjective importance is attached to a skill or opinion. Similarly, Morina (2021) emphasized that social comparisons occur more frequently in areas of life that are particularly important for self-evaluation eliciting stronger emotional and behavioral effects. Accordingly, it is likely that individuals for whom career goals are of high importance react more sensitively to negative feedback and perceive resulting discrepancies more strongly (Hu et al., 2018). This raises the question: To what extent does career centrality influence the relationship between the intensity of LinkedIn use and career-related social comparison? Although theory suggests that individuals who attach greater importance to their careers may respond more strongly to career-related information, empirical evidence regarding the moderating role of career centrality in this specific context is currently lacking. Therefore, we refrained from formulating a directional moderation hypothesis and instead treated this question as exploratory. Whether and how career centrality is involved in this relationship has not yet been studied empirically. Therefore, this research question is considered exploratory.

## 4. Method

### 4.1. Design and Procedure

To investigate the hypotheses, a cross-sectional survey was conducted. Data collection took place via the web-based platform Unipark over a two-week period (mid- to late January 2026). The average time required to complete the questionnaire was estimated at 7 to 12 min. The research project was pre-registered on the Open Science Framework (OSF) platform before the end of data collection.

At the beginning of the online survey, participants received information regarding the anonymity and voluntary nature of the survey, as well as their rights regarding the collected data. Participation was only possible after active consent and confirmation of the inclusion criteria (minimum age of 18 and use of LinkedIn). This was followed by generating a test subject code, and, optionally, by generating a SONA code for psychology students. Furthermore, demographic data were collected, including gender, age, and highest level of education. Regarding occupational information, employment status, and the amount of time spent working were queried. Additionally, participants indicated their frequency of LinkedIn use per week.

The main section of the questionnaire comprised 63 items and one control item. All variables were measured using self-report scales. The survey assessed the intensity of LinkedIn use, career-related social comparison, career centrality, career goal discrepancy, and the revision of career goals. Since this was a survey in German, all scales that were originally in English were translated. The control item was placed roughly in the middle of the survey to identify inattentive and arbitrary response patterns. The order of items was identical for all participants. Apart from the demographic information, all items had to be answered in order to complete the questionnaire.

### 4.2. Sample

The target group for the study was adults who use LinkedIn. Participants were recruited through the researchers’ personal network, via social media platforms such as LinkedIn and Instagram, and through the research platform SurveyCircle. In addition, the survey was posted on the Ruhr University Bochum study page.

A total of *N* = 199 individuals participated in the survey. To ensure good data quality, the data sets were screened according to the selection criteria defined in the pre-registration. Individuals who had not given their consent to participate, who did not meet the inclusion criteria of being of legal age and using LinkedIn, and who did not answer the control item for the attention check correctly were excluded. In addition, individuals who exhibited unusual response patterns, such as overly consistent responses across multiple scales and a conspicuously short processing time of less than 2 min and 30 s, were removed from the sample. Incomplete data sets were automatically filtered by the survey software. After applying these criteria, the final sample size was *N* = 188.

Of these, 60.6% (*n* = 114) were female, 38.8% (*n* = 73) were male, and 0.5% (*n* = 1) identified as non-binary. The participants’ ages ranged from 19 to 58 years, with a mean of 26.51 years (*SD* = 5.95). The highest level of education was distributed as follows: vocational diploma/A-levels (29.3%, *n* = 55), vocational training (4.8%, *n* = 9), bachelor’s degree (36.7%, *n* = 69), master’s degree/diploma/state examination/magister (25.0%, *n* = 47), and doctorate/habilitation (4.3%, *n* = 8). At 68.1% (*n* = 128), the majority of participants were students, while 28.2% (*n* = 53) were employed and 1.6% (*n* = 3) were self-employed. 1.6% (*n* = 3) stated that they were job seekers, and 0.5% (*n* = 1) reported being in a different employment situation. With respect to current employment status, 17.6% (*n* = 33) were not employed, 17.6% (*n* = 33) were in marginal employment, 27.7% (*n* = 52) were working students, 12.2% (*n* = 23) were employed part-time (20 to 34 h per week), and 25% (*n* = 47) were employed full-time (35 or more hours per week). The majority of respondents used LinkedIn for less than one hour per week (*n* = 79).

### 4.3. Measures

The intensity of LinkedIn use was measured using a questionnaire developed by Ozimek et al. (2026). The scale measures the level of activity on LinkedIn using 24 items on a 5-point Likert scale ranging from 1 (never) to 5 (very frequently) (see Table A1). The items are distributed across six subscales, and an overall score was calculated to reflect the general intensity of LinkedIn use. The scale has already been validated for use with the professional network XING and demonstrated very good internal consistency with α = 0.92 (Brandenberg et al., 2019). An example item is: “I search for people I know or might know.”

To measure career-related social comparison, an adapted version of the social comparison questionnaire by Schneider and Schupp (2011) was used, which is the German version of the Iowa-Netherlands Comparison Orientation Measure (INCOM) by Gibbons and Buunk (1999). The scale measures an individual’s tendency to compare themselves with others. For the present study, the wording of the items was adapted to the career context so that the extent to which individuals compare their careers and career success with others was measured. For example, the item “I am not the type of person who often compares myself with others” was adapted into the career-related item “I am not the type of person who often compares my career success with others.” The scale consists of 11 items distributed across the two subscales Comparison of Abilities and Comparison of Opinions. Two items are reverse-scored. Agreement with the statements was measured on a 5-point Likert scale ranging from 1 (Strongly disagree) to 5 (Strongly agree).

An exploratory factor analysis (EFA) was conducted (*N* = 188) to examine the two-factor structure of the adapted scale. Model fit was assessed using several fit indices: The χ^2^ test evaluates the model’s fit to the data, with a non-significant result indicating a good fit, though this is rare with larger samples (Bentler & Bonett, 1980). In addition, the Root Mean Square Error of Approximation (RMSEA; acceptable fit at ≤0.08) and the Tucker–Lewis Index (TLI; acceptable fit at ≥0.95) were used (Moosbrugger & Kelava, 2020). The two-factor model showed an acceptable fit (χ^2^(34) = 62.10, *p* < 0.01, RMSEA = 0.04, TLI = 0.95) and thus corresponded to the structure of the original German scale. As with the original scale, Item 11 (reversed) loaded on the Abilities factor rather than the theoretically postulated Opinions factor. An overview of the item wording and factor loadings can be found in Table A2. For the entire unmodified German scale, a good internal consistency of α = 0.85 was reported (Ozimek & Bierhoff, 2020). In addition, a comparable adaptation for measuring career-related social comparison was used by Fukubayashi and Fuji (2021) in a Japanese sample, demonstrating an excellent reliability of α = 0.95 for the Abilities subscale and α = 0.94 for the Opinions subscale.

Career centrality was measured using a scale developed by Erdogan et al. (2018). The English scale was translated into German and validated using a back-translation procedure (Brislin, 1970) (see Table A3). It consists of three items (e.g., “My career is very important to me”), which are rated on a 6-point scale ranging from 1 (strongly disagree) to 6 (strongly agree). Higher scores indicate that the individual places a high value on their career. An EFA (N = 188) revealed a clear one-factor structure with high factor loadings (λ = 0.66–0.85), with the factor explaining 56% of variance. The internal consistency of the original scale was α = 0.80 (Erdogan et al., 2018).

The career goal discrepancy was measured using the 12-item scale by Creed and Hood (2015). It indicates the perceived gap between an individual’s career goal and their current progress. Higher scores indicate a greater perceived negative discrepancy. Items were rated on a 6-point Likert scale ranging from 1 (Strongly disagree) to 6 (Strongly agree). For this study, the items were translated into German, and their structure was examined using an EFA (*N* = 188, see Table A3). The originally unidimensional factor structure could not be replicated in the German version. Instead, a two-factor structure was revealed. One item (No. 4) exhibited a dual factor loading and was consequently excluded. The final two-factor model showed a good model fit (χ^2^(34) = 15.75, *p* = 0.002, RMSEA = 0.03, TLI = 0.97). The original scale addresses four domains of the discrepancy: achievement (e.g., “My plans are not working out to get the career I really want.”), effort (e.g., “I am working hard, but still doubt I will end up with the career I would really like.”), standard (e.g., “I have an image of my dream job, but I think it is out of my reach.”), and ability (e.g., “I am not sure I am capable of meeting the requirements for the career I really want.”). In the EFA, the three items on achievement discrepancy (Items 1–3) formed an independent first factor, while the remaining dimensions of discrepancy (effort, standard, ability; Items 5–12) were assigned to Factor 2. A Cronbach’s α = 0.95 was reported for the original scale (Creed & Hood, 2015).

The direction of career goal revision was first measured using a single item on a 10-point scale. The item was: “To what extent do you intend to adjust your career goals in the near future?” The scale endpoints were labeled 1 (I will set easier career goals) and 10 (I will set more ambitious career goals). This item served, on the one hand, as external validation for the two scales on goal revision and, on the other hand, provided a baseline for the direction of goal revision. On average, participants reported that they intend to set more ambitious career goals (*M* = 7.06, *SD* = 1.77).

To measure the intention to revise one’s career goals upward in a more nuanced way, an adapted version of the questionnaire by Hu et al. (2017) was used. The original scale measures downward goal revision in a career context. Here, the items were reversed so that they reflected upward adjustment (i.e., setting more ambitious goals) and were translated into German (see Table A4). Responses were given on a 6-point Likert scale ranging from 1 (Strongly disagree) to 6 (Strongly agree). A previous study in which the scale was adapted in the same way reported very good internal consistency (α = 0.88 at time point 1; α = 0.91 at time point 2; Akmal et al., 2022). The scale consists of six items (e.g., “I intend to pursue a more challenging career goal than the one I currently have.”), which were introduced with the following instruction: “The following statements refer to the extent to which you intend to raise your career goals,” with the word “raise” highlighted. The construct validity of the scale is supported by the significant positive correlation (*r* = 0.34, *p* < 0.001) with the single directional item.

To measure the downward revision of career goals, the scale developed by Hu et al. (2017) was employed which measures the intention to lower one’s career goals and pursue less ambitious goals. All six items were translated and adopted without any changes to their content (see Table A3). The instruction read: “The following statements refer to the extent to which you intend to lower your career goals”; the keyword “lower” was highlighted. In previous studies, the scale demonstrated good internal reliability (α = 0.88 at time point 1; α = 0.91 at time point 2; Hu et al., 2017). For downward revision, the correlation with the individual item provides evidence of good construct validity as well (*r* = −0.23, *p* < 0.01).

Exploratory factor analyses were conducted separately for both directions of revision, as well as for a combined two-factor model (*N* = 188). The model, which represented the overall intention of revision across the two factors regardless of direction, exhibited the best, albeit unsatisfactory, model fit (χ^2^(34) = 135.15, *p* < 0.001, RMSEA = 0.05, TLI = 0.90), meaning that the assumed factor structure could not be confirmed in this sample. Since an established scale was available only for downward revision, the revision of career goals in both directions was measured for the first time in this form, and the internal consistency of the scale in this study was satisfactory (α = 0.78); the total scale was employed for the hypothesis tests.

### 4.4. Statistical Analyses

The statistical software RStudio (R Version 4.4.2) was used for data analysis. The required sample size was determined using an a priori power analysis with G*Power 3.1 for multiple linear regression (fixed model, *R*^2^ deviation from zero) based on the most complex hypothesis (H5). Assuming a medium effect size (*f*^2^ = 0.15), a significance level of α = 0.05, a power of 1 − β = 0.95, and three predictors, the required sample size was *N* = 119. To account for potential data loss in accordance with the pre-registered selection criteria, an additional 25% of participants were included in the sample. This resulted in a target sample size of *N* = 150.

Descriptive statistics were calculated for all scales used. An overall mean was calculated for each scale by averaging the item mean scores per person. Higher scores indicate a higher degree of the respective construct. The internal consistency of each scale was determined using Cronbach’s alpha.

To test hypotheses H1–H4, product–moment correlations were calculated. All analyses were performed at a significance level of *p* < 0.05 (two-tailed). The calculation of correlations requires interval-scaled data, linear relationships between the variables, and normal distribution (Bortz & Schuster, 2010; Schäfer, 2016). Due to the use of Likert scales, the data could be treated as quasi-interval scaled. Linearity was assessed visually using scatter plots with a LOESS fit line, while normal distribution was evaluated through a combined examination of histograms, the Kolmogorov–Smirnov test, and the distribution parameters skewness and kurtosis.

To test hypothesis H5, a serial mediation analysis (PROCESS Model 6 following Hayes, 2018) was conducted (*p* < 0.05, two-tailed). The indirect effects were estimated using bootstrapping with 10,000 resamples. The effects tested were considered significant if the 95% confidence interval did not include the value zero. The direction and strength of the relationships were interpreted based on the standardized regression coefficients (β). Since these were estimated within the framework of multiple regression analyses, the relevant model assumptions were tested in advance. Linearity, multicollinearity (using the variance inflation factor), homoscedasticity (using the Goldfeld-Quandt test), and the normal distribution of the residuals were examined using the Kolmogorov–Smirnov test, histograms, and measures of skewness and kurtosis.

For the exploratory analysis of career centrality, a moderation analysis in the form of a multiple regression with an interaction term and a mediation analysis were conducted. The relevant regression assumptions were also tested for these analyses. To improve the interpretability of the coefficients, the relevant variables were z-standardized (*M* = 0, *SD* = 1).

## 5. Results

### 5.1. Descriptive Statistics

Table 1 provides an overview of the scales used and their descriptive statistics. Mean values, standard deviations, intercorrelations, and Cronbach’s alpha are reported. While the scales for career goal revision and career centrality achieve acceptable Cronbach’s alpha values just below 0.8, the scales for LinkedIn use (α = 0.89) and career-related social comparison (α = 0.87) demonstrate good reliability. The scale for career goal discrepancy achieves an excellent internal consistency of α = 0.94. The findings confirm the reliable measurement of the relevant constructs.

On average, participants reported low to moderate LinkedIn use. Regarding career-related comparison behavior, on average there was a moderate tendency toward agreement. The perceived career goal discrepancy was rather low in the overall sample, whereas career centrality was moderate to high. The willingness to revise career goals upward (*M* = 3.6, *SD* = 0.99) was significantly more pronounced than downward (*M* = 2.1, *SD* = 0.89). On average, the overall revision was rather low.

### 5.2. Hypothesis Tests

Before testing the correlation hypotheses, the statistical assumptions were checked for all variables. The assumption of linearity was supported using scatter plots with a LOESS curve. Based on a combined analysis of histograms, Kolmogorov–Smirnov tests, and measures of skewness and kurtosis, it could be assumed that all variables followed an approximately normal distribution.

As hypothesized in H1, there was a significant positive correlation between the intensity of LinkedIn use and the revision of career goals, *r* = 0.24, *p* < 0.001. More intensive use of LinkedIn is associated with a stronger intention to revise one’s own career goals. The correlation coefficient indicates a moderate effect.

In accordance with H2, a significant positive correlation was found between the intensity of LinkedIn use and career-related social comparison, *r* = 0.35, *p* < 0.001. This moderate effect suggests that individuals who use LinkedIn more frequently and to a greater extent engage in career-related comparisons.

H3 postulated a positive relationship between career-related social comparison and perceived career goal discrepancy. The significant positive correlation between the two scales supported this hypothesis, *r* = 0.33, *p* < 0.001. Accordingly, individuals with more pronounced career-related comparison behavior perceive greater discrepancies between their current career status and their desired career goals.

In line with H4, a significant positive relationship was found between perceived career goal discrepancy and the revision of career goals, *r* = 0.50, *p* < 0.001. Higher levels of perceived career goal discrepancy are associated with a stronger intention to revise career goals. H4 was thus supported with a large effect size.

H5 specified a serial mediation model, according to which the relationship between LinkedIn use and the revision of career goals is mediated by career-related social comparison and perceived career goal discrepancy. The prerequisites for mediation analyses were tested and supported, with the exception of the normal distribution of the residuals.

**Figure 2:**
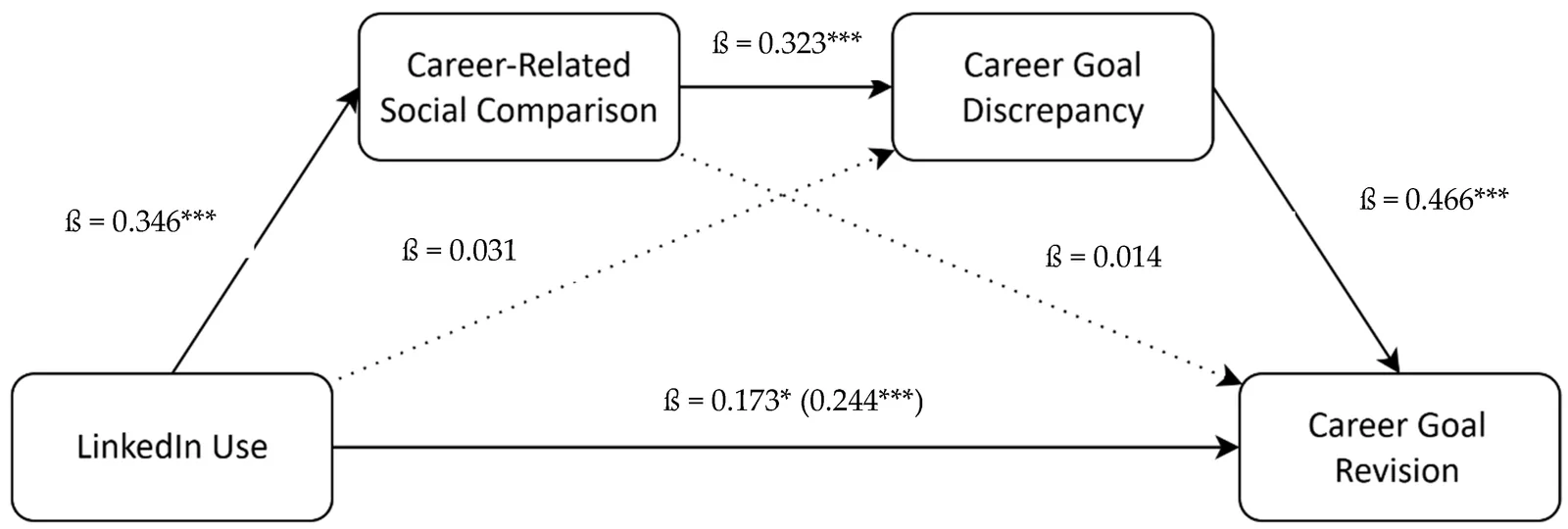
Serial mediation model for predicting the revision of career goals through LinkedIn use, career-related social comparison, and career goal discrepancy. Note. *N* = 188, β = standardized regression coefficients, dashed lines indicate non-significant paths (*p* > 0.05), * *p* < 0.05, *** *p* < 0.001.

The results of the path analysis (see Figure 2 and Table 2) revealed a significant serial mediation in the expected direction, indirect effect: β = 0.052, BC 95%-KI [0.021, 0.094]; overall model: *F*(3, 184) = 23.45, *p* < 0.001, *R*^2^ = 0.28. The individual paths in the mediation chain proved to be significant. More intensive LinkedIn use was associated with stronger career-related social comparison, which in turn was associated with a higher perceived career goal discrepancy. Finally, a larger career goal discrepancy predicted a significantly stronger revision of career goals in a positive manner. The total effect of LinkedIn use on career goal revision was significant. When controlling for the mediators, this relationship was weakened but remained significant. The specific indirect effects via each individual mediator proved to be insignificant, as the corresponding confidence intervals included the value zero (see Table 2). The overall model explained 28% of variance in the revision of career goals. Since the serial indirect effect was significant and the direct effect of the dependent variable on the criterion remained significant when controlling for the mediators, only partial serial mediation rather than full mediation occurred. Thus, H5 was partly supported.

A moderation analysis was conducted to explore whether career centrality significantly influences the relationship between LinkedIn use and career-related social comparison. The assessment of model assumptions revealed that homoscedasticity was met and no multicollinearity occurred, while the residuals deviated from a normal distribution.

The moderation analysis revealed that career centrality did not significantly moderate the relationship between LinkedIn use and reported comparison behavior (see Table 3). The strength of the relationship between LinkedIn use and career-related social comparison is thus independent of the degree of career centrality. The two main effects were significant. When LinkedIn use was increased by one standard deviation, the reported comparison behavior increased by β = 0.396 standard deviations, assuming average career centrality. Similarly, a significant positive effect of career centrality on career-related comparison could be demonstrated when LinkedIn use was average (β = 0.144, *p* < 0.01). Thus, both higher levels of LinkedIn use and career centrality contribute independently to a higher degree of career-related social comparison.

Alternatively, it was examined whether career centrality mediates the relationship between LinkedIn use and career-related social comparison. There was no significant effect of LinkedIn use on career-related social comparison via career centrality (β = 0.027, 95% CI [−0.009, 0.091], *p* = 0.280), indicating that mediation did not occur. The intensity of LinkedIn use did not significantly predict career centrality (β = 0.185, *p* = 0.210, 95% CI [−0.099, 0.486]). When controlling for LinkedIn use, a significant positive relationship was found between career centrality and career-related social comparison (β = 0.145, *p* = 0.007, 95% CI [0.038, 0.247]). The total effect of LinkedIn use on social comparison remained significant (β = 0.427, *p* < 0.001, 95% CI [0.261, 0.610]).

### 5.3. Post Hoc Analyses

**Figure 3:**
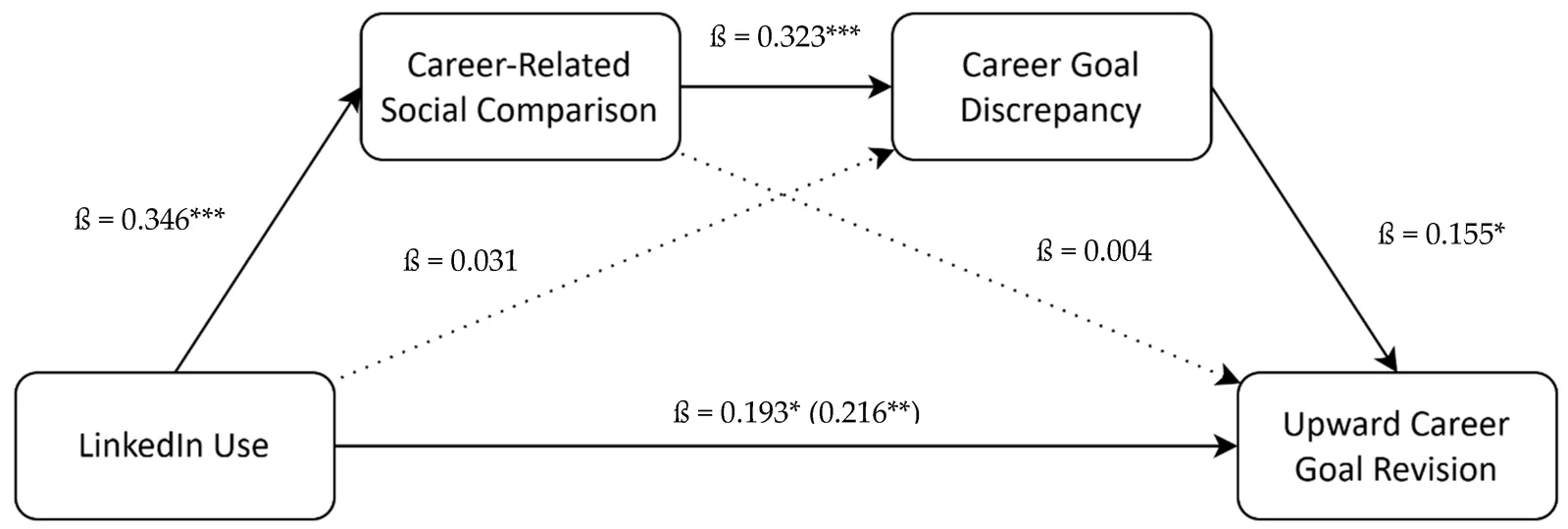
Serial mediation model for predicting upward career goal revision through LinkedIn use, career-related social comparison, and career goal discrepancy. Note. *N* = 188, β = standardized regression coefficients, dashed lines indicate non-significant paths (*p* > 0.05), * *p* < 0.05. ** *p* < 0.01. *** *p* < 0.001.

Since the primary analyses did not distinguish between upward and downward goal revision, no conclusions can be drawn regarding the effect of the predictors on the direction of adjustment. A differentiated analysis of both directions therefore seems relevant to reveal the variation in mechanisms of action. In a post hoc analysis, it was thus exploratively examined whether different patterns of association emerge for upward and downward revision of career goals. For this purpose, both directions of revision were entered as criteria into a serial regression model. Once again LinkedIn use served as the independent variable, whereas career-related social comparison and career goal discrepancy served as mediators. The examination of the assumptions revealed that neither multicollinearity nor heteroscedasticity occurred. The residuals were not normally distributed. The results of the mediation model for upward career goal revision are depicted in Figure 3.

No significant serial indirect effect of LinkedIn use on the upward revision of career goals via career-related social comparison and career goal discrepancy was found (β = 0.017, 95% CI [−0.001, 0.044]; overall model: *F*(3, 184) = 4.64, *p* < 0.01, *R*^2^ = 0.07). Thus, there is no evidence of serial mediation. Nevertheless, the individual paths of association were significant. LinkedIn use positively predicted career-related social comparison, which in turn significantly and positively influenced career goal discrepancy. In addition, a significant positive relationship was found between career goal discrepancy and upward career goal revision. There was a positive relationship between LinkedIn use and upward career goal revision, which remained significant even when the mediators were included, total effect: β = 0.216, *p* < 0.01, 95% CI [0.125, 0.599]. More intensive LinkedIn use is thus associated with a stronger tendency to revise one’s career goals in an upward direction, although not via the mediation chain examined.

**Figure 4:**
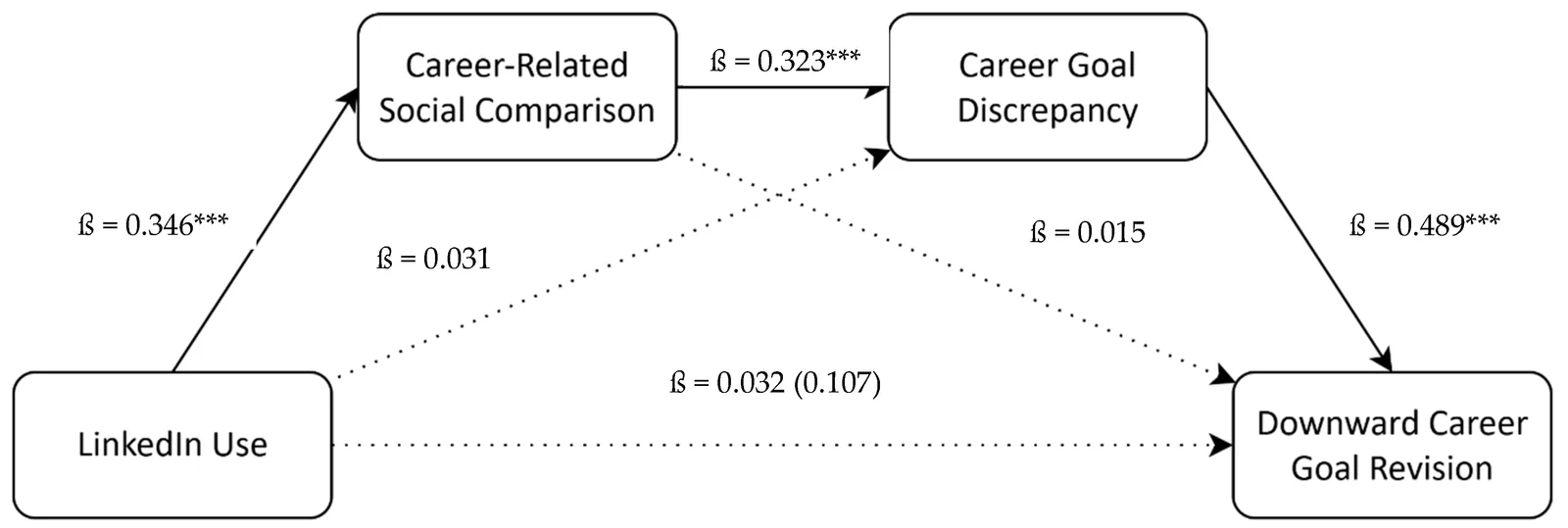
Serial mediation model for predicting downward career goal revision through LinkedIn use, career-related social comparison, and career goal discrepancy. Note. *N* = 188, β = standardized regression coefficients, dashed lines indicate non-significant paths *p* < 0.01. *** *p* < 0.001.

A significant serial mediation was found for the downward revision of career goals, indirect effect: β = 0.055, 95% CI [0.023, 0.097]; overall model: *F*(3, 184) = 20.47, *p* < 0.001, *R*^2^ = 0.25. All associations are displayed in Figure 4. The direct effect of LinkedIn use on downward revision of career goals was not significant (β = 0.032, 95% CI [−0.156, 0.253]), indicating full mediation. Accordingly, LinkedIn use influences the downward revision of career goals exclusively indirectly via career-related social comparison and perceived career goal discrepancy, in that more intensive use leads to stronger comparison behavior, which is associated with a higher perceived discrepancy, which in turn increases the intention to revise career goals downward.

In summary, H1, H2, H3, and H4 were fully supported, while H5 was partially supported.

## 6. Discussion

### 6.1. Summary and Interpretation of Results

The aim of this study was to examine the role of LinkedIn as a context for social comparison in the evaluation and revision of individual career goals. Specifically, we investigated whether the relationship between LinkedIn use and career goal revision is mediated by career-related social comparison and perceived career goal discrepancy. The findings indicate that more intensive LinkedIn use is associated with more frequent career-related social comparison. In turn, stronger comparison behavior was related to greater perceived discrepancies between current career progress and desired career goals. These discrepancies were positively associated with intentions to revise career goals. Together, the results support the proposed serial mediation model and highlight the relevance of social comparison processes for understanding how professional networking platforms influence career-related self-regulation.

Consistent with H1, LinkedIn use was positively associated with career goal revision. This finding extends previous research demonstrating that social media use can influence attitudes and behavioral intentions (Varghese & Agrawal, 2021; Zhao, 2025). While previous studies have primarily focused on specific online activities, such as viewing work-related content (Sender & Korzynski, 2020), the present study suggests that the overall intensity of LinkedIn use is related to the willingness to reconsider and adjust career goals.

The positive association between LinkedIn use and career-related social comparison (H2) is in line with social comparison theory (Festinger, 1954) and previous findings from social media research (Chen et al., 2023; Jang et al., 2016). LinkedIn provides continuous access to information about the professional achievements of others and therefore represents a particularly salient environment for career-related comparisons. Given that users are often connected to individuals with similar educational and occupational backgrounds, the platform offers numerous opportunities for evaluating one’s own career progress relative to relevant comparison targets.

The findings also support H3, indicating that stronger career-related social comparison is associated with greater perceived career goal discrepancy. This result is consistent with the assumption that social comparisons function as a source of feedback by providing information about one’s current standing relative to personal goals and standards (McIntyre & Eisenstadt, 2011; Widyowati et al., 2024). The results therefore suggest that comparisons with others could increase awareness of gaps between desired and actual career development.

In line with H4, perceived career goal discrepancy emerged as a strong correlate of career goal revision. The observed association is comparable to previous findings showing that discrepancies between current and desired states serve as regulatory signals that motivate goal adjustment (Donovan & Hafsteinsson, 2006; Praskova & McPeake, 2022; Theobald et al., 2025). Individuals who perceive larger discrepancies appear more likely to reconsider the attainability of their goals and adjust them accordingly.

The serial mediation model (H5) provided additional insight into the mechanisms underlying these relationships. More intensive LinkedIn use was associated with stronger career-related social comparison, which was linked to greater career goal discrepancy and, ultimately, to greater career goal revision. This finding extends previous research by identifying career-related social comparison as a key mechanism through which LinkedIn use could shape perceptions of career progress influencing subsequent goal regulation. These findings also extend Social Comparison Theory by suggesting that professional networking platforms provide a particularly salient context in which social comparison directly informs career self-regulation. Unlike general social networking sites, LinkedIn predominantly presents career-related comparison standards, making social comparison especially relevant for the evaluation and adjustment of career goals. Our findings therefore highlight the importance of considering the specific characteristics of digital professional environments when applying social comparison theory to career development.

The post hoc analyses revealed important differences between upward and downward career goal revision. For downward revision, the proposed mediation chain was fully supported, indicating that LinkedIn use was related to lowering career goals only indirectly through comparison processes and perceived discrepancies. This finding suggests that downward revision primarily reflects a response to perceived difficulties in attaining desired career outcomes.

In contrast, upward revision was directly associated with LinkedIn use but was not explained by the proposed mediation chain. This pattern suggests that upward and downward goal revision may be driven by partly different psychological mechanisms. Whereas downward revision appears to emerge from comparison-induced perceptions of insufficient progress, upward revision may be influenced by factors such as inspiration, achievement motivation, or career-related self-efficacy. LinkedIn may therefore serve a dual role: it can encourage more ambitious career aspirations while simultaneously contributing to downward goal adjustment when social comparisons foster perceptions of discrepancy.

An important implication of these findings is that upward and downward career goal revision may be better conceptualized as distinct constructs rather than opposite ends of a single continuum. Although the present study employed an overall measure of career goal revision for the preregistered analyses, the post hoc findings suggest that combining both directions may have obscured meaningful differences in their underlying psychological mechanisms. Future research should therefore examine upward and downward career goal revision separately and further validate their distinct measurement and theoretical foundations.

Finally, career centrality neither moderated nor mediated the relationship between LinkedIn use and career-related social comparison. Although individuals with higher career centrality reported engaging in more career-related social comparison overall, career centrality did not strengthen the association between LinkedIn use and career-related social comparison. One possible explanation is that LinkedIn’s highly standardized, success-oriented environment may encourage career-related social comparison regardless of how central an individual’s career is to their self-concept. Because users are routinely exposed to highly curated indicators of professional success, the platform itself may provide sufficiently salient comparison cues to trigger comparison processes across users with different levels of career centrality. Future research should investigate this possibility by examining whether platform characteristics outweigh individual differences in determining comparison behavior.

Overall, the findings suggest that LinkedIn is more than a platform for networking and career development. By increasing exposure to the professional achievements of others, it creates a context in which users evaluate their own career progress, perceive discrepancies between current and desired outcomes, and reconsider their career goals. Career-related social comparison and career goal discrepancy therefore appear to represent important mechanisms linking LinkedIn use to career goal revision. At the same time, the proposed model explained only part of the variance in career goal revision (28%), indicating that additional psychological, situational, and contextual factors are likely to contribute to career goal adjustment. The present findings should therefore be understood as identifying one important mechanism rather than providing a comprehensive explanation of career goal revision.

### 6.2. Limitations

Several limitations should be considered when interpreting the findings. First, the study employed a cross-sectional design, which does not allow causal conclusions to be drawn. Although the serial mediation model is theoretically plausible and consistent with the data, the observed relationships should be interpreted with caution. Longitudinal or experimental designs are needed to confirm the temporal ordering of the proposed mechanisms and to rule out a reverse pathway, whereby individuals who are already reconsidering their career goals turn to LinkedIn more intensively.

Second, several measurement instruments were translated and adapted for the present study. While all scales demonstrated acceptable to excellent internal consistency, the expected factor structures could not be fully replicated for the Career Goal Discrepancy Scale and the Career Goal Revision Scale. Future research should further validate these German adaptations of the scales and examine their dimensionality in larger and more diverse samples. In this study, a single composite score was used to measure intensity of LinkedIn use, aggregating responses across different platform activities without allowing conclusions about which specific behaviors (e.g., viewing profiles or posting) drive the observed effects. Rather than overall usage intensity, it may be specific activities that are more strongly associated with career-related social comparison—a possibility that future research could examine.

Third, we used exploratory factor analyses to assess the construct validity of the instruments. A confirmatory factor analysis (CFA) is the preferred method because it is hypothesis-driven, but it could not be conducted within the scope of this study because EFA and CFA were to be calculated in two separate samples, and because the statistical power was insufficient (Kline, 2016; Worthington & Whittaker, 2006). Note that although the translated scales showed satisfactory internal consistency, some of the expected factor structures could not be replicated. Therefore, the present findings should be interpreted as preliminary evidence, and future research should further validate the German versions of these instruments using independent samples and confirmatory factor analyses.

Fourth, all variables were assessed using self-report questionnaires. Although anonymous participation and the use of established multi-item scales may have reduced evaluation apprehension and response bias, the observed associations may nevertheless be influenced by common method variance, response styles, or social desirability. Future research should therefore combine self-report measures with behavioral indicators, informant reports, or longitudinal designs and consider statistical procedures to assess common method variance.

Finally, the sample consisted predominantly of young adults and university students who were at relatively early stages of their careers. As career goals and career decision-making processes may differ across career stages, the generalizability of the findings is limited. Replication with older and more professionally experienced samples is therefore recommended. Furthermore, participants reported relatively low levels of LinkedIn use on average. Although significant effects were found despite this restricted range, stronger associations may emerge among more active users of professional networking platforms.

### 6.3. Implications for Theory and Future Research

The present study contributes to the growing literature on social comparison in digital career contexts by identifying career-related social comparison and career goal discrepancy as mechanisms linking LinkedIn use to career goal revision. While the findings offer new insights into these processes, they should be regarded as an initial exploration that requires further empirical investigation. There are several directions for future research.

Most importantly, future studies should examine these relationships using longitudinal or experimental designs to clarify the causal dynamics underlying career goal revision. Experimental studies manipulating exposure to different types of LinkedIn content (e.g., highly idealized versus more realistic career-related information) could provide further insights into how online environments shape social comparison and career-related perceptions. The post hoc findings suggest that upward and downward career goal revision may be driven by different psychological mechanisms. While downward revision was explained by the proposed mediation chain, upward revision was not. Future research might investigate additional factors that may promote upward goal revision, such as career-related self-efficacy, achievement motivation, or other positive self-regulatory processes.

Furthermore, future research could extend the present model by considering additional individual difference variables, such as career-related self-efficacy, self-esteem, personality traits, goal orientation, or resilience, which may influence how individuals respond to career-related social comparisons on professional networking platforms.

Additionally, future studies may benefit from distinguishing between positive and negative forms of perceived discrepancy. Whereas the present study focused on negative discrepancies, positive discrepancies may represent an alternative pathway through which social comparisons encourage more ambitious career goals. Examining both pathways simultaneously could contribute to a more differentiated understanding of career goal revision in professional online environments.

Finally, future research should investigate whether the results vary across different career stages. Because career goals, opportunities, and developmental tasks change throughout the lifespan, the effects of LinkedIn use and career-related social comparison may differ substantially between early-career and more experienced professionals.

### 6.4. Practical Implications

Our findings suggest that LinkedIn use is associated with career-related social comparison and the evaluation of personal career progress. Consequently, users should be aware that professional networking platforms not only provide information and networking opportunities but may also influence how they perceive their own careers and future aspirations.

Particularly for individuals who frequently compare themselves with others, it may be beneficial to critically reflect on the highly selective and achievement-focused nature of professional online profiles. Recognizing that LinkedIn primarily highlights successes rather than setbacks may help users place social comparisons into a more realistic perspective and reduce unnecessary pressure.

The results also underline the importance of regularly reflecting on personal career goals and the standards used to evaluate career progress. Because perceived discrepancies were strongly associated with career goal revision, individuals may benefit from assessing whether their goals remain realistic, meaningful, and aligned with their personal values rather than relying primarily on comparisons with others.

The findings also extend to broader organizational contexts, including employer branding, career counseling, and talent management. Organizations can use authentic career-related content, such as employee testimonials or behind-the-scenes insights, to provide more realistic information about career trajectories and reduce unrealistic expectations. Career counseling and mentoring initiatives can further support individuals in critically reflecting career comparisons, adapting their goals constructively, and developing sustainable career pathways. Such practices may strengthen career orientation and contribute to employee retention. Apart from LinkedIn, in-person networking opportunities, such as career fairs or professional events, may also support more realistic perceptions of career development. By providing the opportunity to ask questions and learn from one another, professional developments can be viewed in a more realistic light, which may help individuals to focus less on the idealized career paths presented on social media.

Beyond career counseling, these findings may also be relevant for organizations and human resource professionals. Because professional networking platforms increasingly shape employees’ perceptions of career success and advancement, HR development initiatives could encourage a more reflective use of LinkedIn, promote realistic career expectations, and support employees in interpreting career-related social comparisons in a constructive rather than discouraging way.

Overall, the findings emphasize that professional networking platforms play a meaningful role in career development by shaping how individuals evaluate their progress and adjust their career goals.

## Figures and Tables

**Table 1:** Cronbach’s alpha, means, standard deviations, and correlations of the variables examined.

Variable	α	*M*	*SD*	1	2	3	4
1. Intensity of LinkedIn Use (1–5)	0.89	2.56	0.59	–			
2. Career-Related Social Comparison (1–5)	0.87	3.23	0.73	0.35 ***	–		
3. Career Goal Discrepancy (1–6)	0.94	2.88	1.08	0.14	0.33 ***	–	
4. Career Goal Revision (1–6)	0.78	2.85	0.63	0.24 ***	0.23 **	0.50 ***	–
5. Career Centrality (1–6)	0.79	3.54	0.99	0.11	0.23 **	0.12	0.09

Note. *N* = 188, the range of values for each scale is indicated in brackets, *M* = mean, *SD* = standard deviation, product–moment correlation (two-tailed), ** *p* < 0.01. *** *p* < 0.001.

**Table 2:** Indirect effects of the mediation model for predicting the revision of career goals through LinkedIn use, career-related social comparison, and career goal discrepancy.

Indirect Path	β	*Boot SE*	95% CI	
			*LL*	*UL*
Total indirect effect	0.072	0.044	−0.015	0.159
a_1_b_1_ LinkedIn → CRSC → GR	0.005	0.026	−0.050	0.056
a_2_b_2_ LinkedIn → CGD → GR	0.015	0.036	−0.058	0.085
a_1_d_21_b_2_ LinkedIn → CRSC → CGD → GR	0.052	0.019	0.021	0.094

Note. *N* = 188, β = standardized regression coefficients, *Boot SE* = bootstrap standard error, CI = confidence interval, *LL* = lower limit, *UL* = upper limit, LinkedIn = intensity of LinkedIn use, CRSC = career-related social comparison, CGD = career goal discrepancy, GR = career goal revision.

**Table 3:** Results of the multiple regression analysis with career-related social comparison as the criterion, LinkedIn use as the predictor, and career centrality as the moderator.

Path	β	*SE*	*t*	*p*
Constant	−0.003	0.049	−0.052	0.958
Intensity of LinkedIn Use	0.396	0.084	4.698	<0.001
Career Centrality	0.144	0.050	2.892	0.004
Interaction	0.041	0.071	0.569	0.570

Note. *N* = 188, β = standardized regression coefficients, *SE* = standard error, *p* < 0.05 is considered significant, significant results are highlighted, *F*(3, 184) = 11.68, *p* < 0.001, *R*^2^ = 0.16.

## Data Availability

The dataset generated and analyzed during the current study is available in the OSF repository: https://osf.io/pn9b7/overview?view_only=862dac4adfcc4f26ad735f6b8608bb4b (accessed on 17 July 2026).

## Appendix A. Item Overview with Descriptive Statistics

**Table A1:** Descriptive statistics for the scale Intensity of LinkedIn Use.

Item	English	German	*M*	*SD*
1	I participate in discussions about posts in the news feed.	Ich nehme an Diskussionen über Beiträge im Nachrichtenbereich teil.	1.29	0.58
2	I create or comment on posts in groups or forums.	Ich erstelle oder kommentiere Beiträge in Gruppen oder Foren.	1.37	0.65
3	I share news updates.	Ich teile Neuigkeiten.	1.97	0.93
4	I comment on other users’ status updates.	Ich kommentiere Status-Updates anderer Nutzer:innen.	1.97	0.96
5	I check whether my private messages have been read or not.	Ich überprüfe, ob meine privaten Nachrichten gelesen wurden oder nicht.	2.56	1.23
6	I look at other users’ contacts and networks.	Ich sehe mir die Kontakte und Netzwerke anderer Nutzer:innen an.	3.16	1.17
7	I look at other users’ profile details (e.g., work experience, education, organizations, interests, personal information).	Ich sehe mir die Profildetails anderer Nutzer:innen an (z. B. Berufserfahrung, Ausbildung, Organisationen, Interessen, persönliche Informationen).	3.70	1.01
8	I look at other users’ (recent) activities.	Ich schaue mir die (neuesten) Aktivitäten anderer Nutzer:innen an.	3.10	1.06
9	I look at other users’ groups.	Ich sehe mir die Gruppen anderer Nutzer:innen an.	1.69	0.98
10	I look at what I have in common with other users.	Ich schaue, welche Gemeinsamkeiten ich mit anderen Nutzer:innen habe.	2.57	1.16
11	I follow other users’ profiles.	Ich folge anderen Nutzer:innenprofilen.	3.43	1.07
12	I pay attention to my contacts’ upcoming birthdays.	Ich achte auf bevorstehende Geburtstage meiner Kontakte.	1.74	0.95
13	I send birthday wishes or greeting cards suggested by LinkedIn.	Ich sende Geburtstagswünsche oder Glückwunschkarten, die von LinkedIn vorgeschlagen werden.	1.53	0.87
14	I read articles on the homepage.	Ich lese Artikel auf der Startseite.	3.16	1.20
15	I write and/or read private messages.	Ich schreibe und/oder lese private Nachrichten.	2.75	1.07
16	I look at company profiles.	Ich sehe mir Unternehmensprofile an.	3.24	1.10
17	I look at my profile statistics.	Ich schaue mir meine Profil-Statistiken an.	2.44	1.20
18	I search for jobs (using the regular search function and viewing job recommendations).	Ich suche nach Jobs (reguläre Suchfunktion; Betrachtung von Jobempfehlungen).	2.90	1.29
19	I organize my job search (saved, in progress, applied, archived).	Ich organisiere meine Jobsuche (Gespeichert, in Bearbeitung, Beworben, Archiviert).	2.20	1.28
20	I use my LinkedIn data for my job applications.	Ich nutze meine Daten von LinkedIn für meine Bewerbungen.	2.14	1.18
21	I look at my own profile details.	Ich sehe mir meine eigenen Profildetails an.	3.18	1.09
22	I check whether my profile picture presents me in the best possible light and edit it if necessary.	Ich überprüfe, ob mein Profilbild mich im besten Licht präsentiert und bearbeite es, falls nötig.	2.68	1.23
23	I search for people I know or might know.	Ich suche nach Personen, die ich kenne oder kennen könnte.	3.55	1.03
24	I look at the profiles of people who viewed my profile.	Ich sehe mir die Profile meiner Profilbesucher:innen an.	3.12	1.28

**Table A2:** Scale adaptation and descriptive statistics for the scale Career-Related Social Comparison.

	Career-Related Social Comparison					
Item	English	German	Modified Item	Factor Loading	*M*	*SD*
	*Comparison of Abilities*					
1	I often compare how my loved ones (boy or girlfriend, family members, etc.) are doing with how others are doing.	Ich vergleiche häufig das Wohlergehen meiner Angehörigen (Partner, Familienangehörige, etc.) mit dem von anderen.	Ich vergleiche häufig das berufliche Wohlergehen meiner Kolleg:innen mit dem von anderen Personen.	0.54	2.55	1.21
2	I always pay a lot of attention to how I do things compared with how others do things.	Ich achte immer sehr stark darauf, wie ich Dinge im Vergleich zu anderen mache.	In Bezug auf meine Karriere achte ich immer sehr stark darauf, wie ich Dinge im Vergleich zu anderen mache.	0.78	3.09	1.10
3	If I want to find out how well I have done something, I compare what I have done with how others have done.	Wenn ich herausfinden möchte, wie gut ich etwas erledigt oder gemacht habe, dann vergleiche ich mein Ergebnis mit dem anderer Personen.	Wenn ich herausfinden möchte, wie gut ich berufliche vorankomme, vergleiche ich meinen Karriereerfolg mit dem anderer Personen.	0.83	3.16	1.18
4	I often compare how I am doing socially (e.g., social skills, popularity) with other people.	Ich vergleiche häufig meine sozialen Fähigkeiten und meine Beliebtheit mit denen anderer Personen.	In Bezug auf Karriere vergleiche ich häufig meine sozialen Fähigkeiten und meine Beliebtheit mit denen anderer Personen.	0.58	2.64	1.09
5	I am not the type of person who compares often with others.	Ich bin nicht der Typ Mensch, der sich oft mit anderen vergleicht.	Ich bin nicht der Typ Mensch, der seinen Karriereerfolg oft mit anderen vergleicht.	0.68	3.19	1.10
6	I often compare myself with others with respect to what I have accomplished in life.	Ich vergleiche mich häufig selbst mit anderen in Bezug auf das, was ich im Leben (bislang) erreicht habe.	Ich vergleiche mich häufig selbst mit anderen in Bezug auf das, was ich in meiner Karriere (bislang) erreicht habe.	0.67	3.21	1.12
11	I never consider my situation in life relative to that of other people.	Ich bewerte meine Lebenssituation niemals im Vergleich zu der anderer Personen.	Ich bewerte meinen beruflichen Status niemals im Vergleich zu dem anderer Personen.	0.59	3.59	1.05
	*Comparison of Opinions*					
7	I often like to talk with others about mutual opinions and experiences.	Ich tausche mich gerne häufig mit anderen über Meinungen und Erfahrungen aus.	In Bezug auf Karriere tausche ich mich gerne häufig mit anderen über Meinungen und Erfahrungen aus.	0.65	3.41	1.17
8	I often try to find out what others think who face similar problems as I face.	Ich versuche häufig herauszufinden, was andere denken, die mit ähnlichen Problemen konfrontiert sind wie ich.	In Bezug auf Karriere versuche ich häufig herauszufinden, was andere denken, die mit ähnlichen Problemen konfrontiert sind wie ich.	0.88	3.54	1.15
9	I always like to know what others in a similar situation would do.	Ich möchte immer gerne wissen wie sich andere in einer ähnlichen Situation verhalten würden.	In Bezug auf Karriere möchte ich immer gerne wissen, wie sich andere in einer ähnlichen Situation verhalten würden.	0.73	3.62	1.06
10	If I want to learn more about something, I try to find out what others think about it.	Wenn ich über etwas mehr erfahren möchte, versuche ich herauszufinden was andere darüber denken oder wissen.	Wenn ich über etwas mehr erfahren möchte, versuche ich herauszufinden was andere darüber denken oder wissen.	0.80	3.51	0.99

Note. The two factors are specified in italics.

**Table A3:** Back-translation procedures and descriptive statistics for the scales Career Centrality, Career Goal Discrepancy, and Downward Career Goal Revision.

	Item	Translation	Back-Translation	Factor Loading	*M*	*SD*
	Career Centrality					
1	My personal success depends largely on my career achievement.	Mein persönlicher Erfolg hängt größtenteils von meinen Karriereerfolgen ab.	My personal success largely depends on my career achievements.	0.66	3.38	1.18
2	Career is the first thing in my life.	Karriere steht in meinem Leben an erster Stelle.	Career comes first in my life.	0.85	2.94	1.29
3	My career is very important to me.	Meine Karriere ist mir sehr wichtig.	My career is very important to me.	0.72	4.31	1.10
	Career Goal Discrepancy					
Achievement discrepancy						
1	My plans are not working out to get the career I really want.	Meine aktuellen Pläne werden vermutlich nicht dazu führen, dass ich die Karriere erreiche, die ich mir wirklich wünsche.	My current plans will probably not lead to me achieving the career I want.	0.77	2.89	1.28
2	What I have achieved to date doesn’t give me confidence that I will reach my career goals.	Das, was ich bisher erreicht habe, gibt mir kein Vertrauen darin, dass ich meine Karriereziele erreichen werde.	What I have achieved so far does not give me confidence that I will be able to achieve my career goals.	0.74	2.61	1.34
3	I am making progress on my career goals, but I don’t think I have achieved enough to get the career I want.	Ich mache Fortschritte bei meinen Karrierezielen, aber ich glaube nicht, dass ich genug erreicht habe, um die Karriere zu bekommen, die ich möchte.	I am making progress toward my career goals, but I do not think I have achieved enough to get the career I want.	0.75	3.10	1.32
4	Despite my best efforts, I think I am going to miss out on my ideal career.	Trotz meiner größten Bemühungen glaube ich, dass ich meine ideale Karriere verpassen werde.	Despite my greatest efforts, I believe that I will miss out on my ideal career.	/	2.58	1.32
Career Goal Discrepancy						
5	Even with my best efforts, I think I will have to settle for something less than my ideal career.	Selbst wenn ich mein Bestes gebe, glaube ich, dass ich mich mit etwas zufriedengeben muss, das weniger meiner idealen Karriere entspricht.	Even if I do my best, I believe that I will have to settle for something that is less than my ideal career.	0.64	2.96	1.36
6	I am working hard, but still doubt I will end up with the career I would really like.	Ich arbeite hart, zweifle aber trotzdem daran, ob ich am Ende die Karriere erreiche, die ich wirklich gerne hätte.	I work hard, but I still doubt whether I will ultimately achieve the career I would really like to have.	0.64	3.39	1.46
7	I doubt I can meet the standards of entry to my ideal career.	Ich bezweifle, dass ich die Anforderungen für den Einstieg in meine ideale Karriere erfüllen kann.	I doubt that I can meet the requirements to enter my ideal career.	0.78	3.09	1.39
8	I have set my sights on a particular career, but I don’t think that I am going to reach it.	Ich habe mir eine bestimmte Karriere vorgenommen, aber ich glaube nicht, dass ich sie erreichen werde.	I have set my sights on a particular career, but I do not think that I will attain it.	0.75	2.67	1.25
9	I have an image of my dream job, but I think it is out of my reach.	Ich habe ein Bild von meinem Traumjob, aber ich glaube, dass er außerhalb meiner Reichweite liegt.	I have an image of my dream job, but I believe that it is out of my reach.	0.82	2.85	1.45
10	I thought I had the ability to get the career I want, but now I am not so sure.	Ich dachte, ich hätte die Fähigkeit, die Karriere zu erreichen, die ich möchte, aber jetzt bin ich mir nicht mehr so sicher.	I thought I had the ability to achieve the career I want, but now I am not so sure anymore.	0.81	2.85	1.46
11	I know the career I want, but don’t think I have what it takes to reach it.	Ich weiß welche Karriere ich möchte, aber glaube nicht, dass ich das nötige Potenzial habe, um sie zu erreichen.	I know which career I want, but I do not believe that I have the necessary potential to achieve it.	0.80	2.47	1.26
12	I am not sure I am capable of meeting the requirements for the career I really want.	Ich bin mir nicht sicher, ob ich in der Lage bin, die Anforderungen für die Karriere zu erfüllen, die ich wirklich möchte.	I am not sure whether I am able to meet the requirements for the career I really want.	0.93	2.85	1.39
	Downward Career Goal Revision					
1	I plan to aim for a career that is less demanding than my current choice.	Ich plane, eine Karriere anzustreben, die weniger anspruchsvoll ist als meine derzeitige Wahl.	I plan to pursue a career that is less demanding than my current choice.	0.81	2.09	1.01
2	I intend to pursue an easier career goal than the one I currently have.	Ich beabsichtige, ein leichteres Karriereziel zu verfolgen als das, das ich derzeit habe.	I intend to pursue an easier career goal than the one I currently have.	0.80	2.14	1.02
3	I need to reduce my aspirations as the occupation I am aiming for is unrealistic.	Ich muss meine Ambitionen reduzieren, da der Beruf, den ich anstrebe, unrealistisch ist.	I need to reduce my ambitions because the profession I am striving for is unrealistic.	0.71	2.26	1.21
4	I plan to aim for a less challenging career than my current choice.	Ich plane, eine weniger herausfordernde Karriere anzustreben als meine derzeitige Wahl.	I plan to pursue a less challenging career than my current choice.	0.86	2.07	1.02
5	I plan to lower my career goal as I am aiming too high.	Ich plane, mein Karriereziel niedriger zu setzen, da ich mir ein zu hohes Ziel gesetzt habe.	I plan to lower my career goal because I have set my sights too high.	0.86	2.12	1.11
6	I intend to pursue a less responsible career than my current choice.	Ich beabsichtige, eine weniger verantwortungsvolle Karriere zu verfolgen als meine derzeitige Wahl.	I intend to pursue a career with less responsibility than my current choice.	0.80	1.98	1.01

Note. The factor loadings for the scales measuring career goal revision refer to a scale measuring overall revision, consisting of the two factors upward revision and downward revision.

**Table A4:** Scale adaptation and descriptive statistics for the scale Upward Career Goal Revision.

	Upward Career Goal Revision					
	Item	Modified Item	Translation	Factor Loading	*M*	*SD*
1	I plan to aim for a career that is less demanding than my current choice.	I plan to aim for a career that is more demanding than my current choice.	Ich plane, eine Karriere anzustreben, die anspruchsvoller ist als meine derzeitige Wahl.	0.82	4.07	1.13
2	I intend to pursue an easier career goal than the one I currently have.	I intend to pursue a harder career goal than the one I currently have.	Ich beabsichtige, ein schwierigeres Karriereziel zu verfolgen als das, das ich derzeit habe.	0.75	3.88	1.25
3	I need to reduce my aspirations as the occupation I am aiming for is unrealistic.	I need to raise my aspirations as the occupation I am aiming for is well within my reach.	Ich muss meine Ambitionen erhöhen, da der Beruf, den ich anstrebe, mich nicht genug herausfordert.	0.67	3.11	1.34
4	I plan to aim for a less challenging career than my current choice.	I plan to aim for a more challenging career than my current choice.	Ich plane, eine herausforderndere Karriere anzustreben als meine derzeitige Wahl.	0.84	3.83	1.27
5	I plan to lower my career goal as I am aiming too high.	I plan to raise my career goal as I am aiming too low.	Ich plane, mein Karriereziel höher zu setzen, da ich mir ein zu niedriges Ziel gesetzt habe.	0.57	2.86	1.32
6	I intend to pursue a less responsible career than my current choice.	I intend to pursue a more responsible career than my current choice.	Ich beabsichtige, eine verantwortungsvollere Karriere zu verfolgen als meine derzeitige Wahl.	0.70	3.76	1.32

Note. The factor loadings refer to a scale measuring overall revision, consisting of the two factors upward revision and downward revision.
